# The Relationship between Carotid Intima-Media Thickness and Ocular Circulation in Type-2 Diabetes

**DOI:** 10.1155/2019/3421305

**Published:** 2019-02-20

**Authors:** Kohei Ichinohasama, Hiroshi Kunikata, Azusa Ito, Masayuki Yasuda, Shojiro Sawada, Keiichi Kondo, Chihiro Satake, Hideki Katagiri, Toru Nakazawa

**Affiliations:** ^1^Department of Ophthalmology, Tohoku University Graduate School of Medicine, Sendai, Japan; ^2^Department of Retinal Disease Control, Tohoku University Graduate School of Medicine, Sendai, Japan; ^3^Department of Metabolism and Diabetes, Tohoku University Graduate School of Medicine, Sendai, Japan; ^4^Department of Advanced Ophthalmic Medicine, Tohoku University Graduate School of Medicine, Sendai, Japan; ^5^Department of Ophthalmic Imaging and Information Analytics, Tohoku University Graduate School of Medicine, Sendai, Japan

## Abstract

**Purpose:**

To compare clinical findings, including ocular blood flow and intima-media thickness (IMT) of the carotid artery, in mild nonproliferative diabetic retinopathy (NPDR) and no diabetic retinopathy (NDR) patients, and to determine risk factors contributing to mild NPDR.

**Methods:**

In 129 subjects (129 eyes) with type-2 diabetes patients and mild NPDR or NDR, standard statistical techniques were used to determine associations between clinical findings, including diabetes duration, blood levels of creatinine and hemoglobin A1c (HbA1c), central macular thickness (CMT; measured with optical coherence tomography), mean blur rate (MBR; measured with laser speckle flowgraphy), and ultrasound-measured carotid IMT.

**Results:**

Diabetes duration, IMT, and CMT were significantly higher in the mild NPDR patients than the NDR patients (*P*=0.004, *P*=0.004, and *P*=0.003, respectively), while conversely, MBR in the overall optic nerve head (MBR-A) was lower in the mild NPDR patients. Furthermore, a logistic regression analysis showed that diabetes duration (OR, 1.11; *P*=0.006), diastolic blood pressure (OR, 0.93; *P*=0.025), heart rate (OR, 1.07; *P*=0.004), IMT (OR, 8.65; *P*=0.005), and CMT (OR, 1.03; *P*=0.007) were independent contributing factors to mild NPDR. Spearman's rank correlation test also showed that IMT was negatively correlated with MBR-A (*P*=0.011).

**Conclusions:**

Increased IMT showed a close association with ocular ischemia in patients with type-2 diabetes and contributed to the presence of mild NPDR. These findings suggest that IMT may be an early biomarker of mild NPDR.

## 1. Introduction

Diabetic retinopathy (DR) is one of the most important causes of adult-onset vision loss worldwide [1]. Diabetes mellitus affects approximately 350 million people [2], and one-third of these people will likely be affected by DR at some point. The treatment of DR has improved, both medically and surgically, but it is still relatively difficult if it progresses to diabetic macular edema (DME) or proliferative DR (PDR), even for specialists [3, 4]. DME is often treated with anti-vascular endothelial growth factor (anti-VEGF), but the disease often recurs, and a significant proportion of patients do not respond to anti-VEGF, affecting clinical success [5]. Moreover, in neovascular glaucoma (NVG), which already has a relatively high prevalence of DME after vitrectomy (approximately 10%), preoperative anti-VEGF therapy can be an additional risk factor [6]. Thus, the best strategy is preemptive treatment before DME, and PDR develop in patients with diabetes. This makes it important to find clinically useful new biomarkers of DR [7], as well as to understand the pathogenesis of DME and PDR.

Diabetes causes three main complications, including DR. All of these complications are caused by microvascular disturbance and are generally thought to arise sequentially, after diabetes has had a duration of more than 10 years as follows: first, neuropathy; second, retinopathy; and third, nephropathy. Reduced nerve conduction velocity was recently reported to show an association with early DR in type-2 diabetes patients [8]. Macrovascular complications, such as myocardial infarction and brain infarction due to atherosclerosis, also occur simultaneously with microvascular complications, but macrovascular complications progress during the early, impaired-glucose-tolerance stage of diabetes. Therefore, impaired glucose tolerance may be a risk factor for early-stage atherosclerosis [9]. Early atherosclerosis is present in patients with type-2 diabetes that have not yet received treatment, and glucose tolerance can also be impaired in these patients [10]. Thus, it may be promising to assess atherosclerosis as a diabetic macrovascular complication, in order to predict the occurrence of DR as a diabetic microvascular complication.

Intima-media thickness (IMT) represents the thickness of the inner layers of a vessel, commonly the carotid artery. IMT increases in atherosclerosis, making it a useful biomarker in hypertension, dyslipidemia, and the macrovascular complications of diabetes. Measuring IMT is a useful technique for screening for diabetic complications, both macrovascular and microvascular, because of its ease of use, ready availability, and noninvasiveness [11]. IMT can be measured with ultrasound, and it has been reported that it is associated with laser speckle flowgraphy (LSFG) parameters, such as blowout time (BOT) [12]. This raises the possibility that IMT may be a biomarker of diabetes complications, including ocular complications, in the early stages. However, the relationship between clinical findings, including IMT and ocular blood flow parameters, in diabetes patients is still unclear. Thus, this study set out to determine whether systemic clinical findings, including IMT, were associated with ocular clinical findings, including measurement of the main LSFG blood flow parameter (i.e., mean blur rate (MBR)), in type-2 diabetes patients with mild nonproliferative diabetic retinopathy (NPDR) or no diabetic retinopathy (NDR), in order to search for new, early biomarkers of mild NPDR [13].

## 2. Materials and Methods

### 2.1. Setting and Design

This study was an institutional, cross-sectional, nonrandomized, and observational case series.

### 2.2. Patients

This study followed previously described methods [13]. All subjects (age: 20–80 years) had type-2 diabetes and either mild NPDR or NDR. Observation took place at Tohoku University Hospital. Baseline ophthalmological characteristics were recorded, including visual acuity, intraocular pressure (IOP), slit-lamp results, and fundus appearance.

Subjects were included if they had diabetes mellitus with HbA1c > 6.5% and ongoing pharmacological treatment for diabetes. They were excluded if they had other types of diabetes, including pancreatic, hepatic, or gestational diabetes, secondary diabetes from endocrine disease, or type-1 diabetes. Other exclusion criteria included current hemodialysis, malignant or inflammatory disease, and chronic respiratory disease, as well as age-related macular degeneration, glaucoma, and any other retinal disease.

An experienced ophthalmologist assessed DR severity in the right eye of each patient based on clinical findings, including indirect ophthalmoscopy and slit-lamp biomicroscopy of the posterior segment, which used *a* + 90 D lens (Volk Optical Inc., Mentor, Ohio, USA) in accordance with the criteria of the Early Treatment of Diabetic Retinopathy Study (ETDRS) [13, 14].

Approval for this study was obtained from our institutional review board (Tohoku University Graduate School of Medicine). All patients provided informed consent for their participation in the study (University Hospital Medical Information Network; UMIN Study ID N.: UMIN000023859). All protocols followed the Declaration of Helsinki (1995: revised in Edinburgh, 2000).

### 2.3. Main Outcome Measures

The significance of differences between the mild NPDR and NDR patients in clinical findings was determined, including diabetes duration, creatinine, blood levels of hemoglobin A1c (HbA_1c_), optical coherence tomography (OCT)-measured central macular thickness (CMT), LSFG-measured optic nerve head (ONH) MBR, and IMT in ultrasound B-scans (obtained in the common carotid artery on the right side). Additionally, we evaluated the associations between these findings with standard statistical techniques.

### 2.4. Clinical and Ophthalmological Examination

Measurements included systolic blood pressure (SBP), diastolic blood pressure (DBP), and heart rate (HR). These measurements were obtained after asking the patients to sit quietly for 10 minutes. The target of all blood flow measurements was the left brachial artery, at the same height as the heart. An automatic blood pressure monitor was used (HEM-759E, Omron Corporation, Kyoto, Japan). The subjects fasted for 12 hours before the blood samples were collected. Standardized, automatic laboratory techniques were used to measure HbA_1c_, total cholesterol, and creatinine. IMT in ultrasound B-scans of the right CCA was measured with the ProSound F75 (Hitachi-Aloka, Tokyo, Japan). Measurements of IMT in this study represented an average of the length between the carotid bulb of the common carotid artery to the internal carotid artery. IMT was defined as the layer between the edge of the first echogenic line (which represents the upper adventitia layer, containing collagen) and the second echogenic line. Additionally, we calculated the maximum value for IMT, including plaque lesions, (i.e., IMT > 1.1 mm) and defined it as IMT-Cmax [15]. We also obtained measurements of visual acuity, IOP, and spherical equivalent (SE) and performed fundus photography. CMT measurements were obtained with OCT (Topcon 3D OCT-2000, Topcon, Tokyo, Japan). Only right eyes were included in this study.

### 2.5. LSFG

LSFG used a measurement method previously described. In brief, the subjects undergo dilation of the pupil with tropicamide (0.5%) and phenylephrine hydrochloride (0.5%) [16, 17]. The LSFG device (Softcare, Fukutsu, Japan) then irradiates the retina with laser light and measures the resulting speckling caused by scattering in the fundus tissue. The light intensity of the speckling is then used as the basis for software calculation of MBR, for each image pixel. The output of the software is a map showing MBR over time in the overall ONH (MBR-A). This map is also divided into separate areas based on the presence of large vessels (MBR-V) or capillaries (i.e., nonvessel tissue; MBR-T). Parameters of the pulse waveform can then be obtained separately in these areas. Of particular interest in this study was blowout time (BOT) [18, 19]. Statistical analyses in this study used three sets of LSFG measurements averaged together.

### 2.6. Statistical Analyses

Variables were expressed as median (interquartile range). Clinical findings were compared in the mild NPDR and NDR groups with the Mann–Whitney *U* test and chi-squared test. Relationships between measurement parameters were estimated with Spearman's rank correlation test. Multiple logistic regression analysis was used to calculate whether the presence of mild NPDR was significant in the patients. The pROC package in R software (version 1.13.0) was used to perform a receiver operating characteristic (ROC) curve analysis to assess the ability of IMT-Cmax to predict mild NPDR. An ROC curve was also used for a logistic regression model including all variables. Statistical analysis used the R software package (v. 3.2.0, R core team). The significance level was set at *P* < 0.05.

## 3. Results

Table 1 shows clinical findings in the subjects. A total of 129 type-2 diabetes patients were included (75 men and 54 women with a median age of 54). Ninety-nine patients (55 men and 44 women with a median age of 54) had NDR, and 30 patients (20 men and 10 women with a median age of 55.5) had mild NPDR. Age, sex, HbA1c, creatinine, SBP, total cholesterol, VA, SE, and IOP were similar in the mild NPDR and NDR groups (*P*=0.68, *P*=0.38, *P*=0.38, *P*=0.92, *P*=0.67, *P*=0.59, *P*=0.18, *P*=0.94  and  *P*=0.996, respectively, Table 1). However, the mild NPDR group had a significantly longer duration of diabetes (*P*=0.004) and significantly higher IMT-Cmax and CMT (*P*=0.004 and *P*=0.003, respectively) compared to the NDR group (Table 1). Furthermore, the mild NPDR group had lower MBR-T and MBR-V compared to the NDR group, even though this did not reach statistical significance (*P*=0.16 and *P*=0.13, respectively). The mild NPDR group had significantly lower MBR-A compared to the NDR group (*P*=0.045). BOT-A, BOT-T, and BOT-V were statistically similar in the mild NPDR and NDR groups (*P*=0.995, *P*=0.65 and *P*=0.86, respectively).

**Figure 1:**
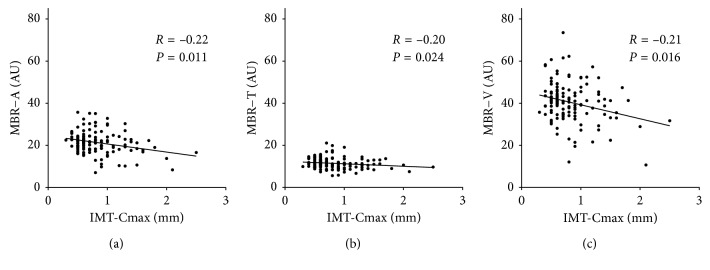
Relationship between IMT and MBR. (a) MBR in the overall optic nerve head was negatively correlated with IMT-Cmax (*R*=−0.22, *P*=0.011). (b) MBR in the optic nerve head tissue area was negatively correlated with IMT-Cmax (*R*=−0.20, *P*=0.024). (c) MBR in the optic nerve head vascular area was negatively correlated with IMT-Cmax (*R*=−0.21, *P*=0.016).

**Figure 2:**
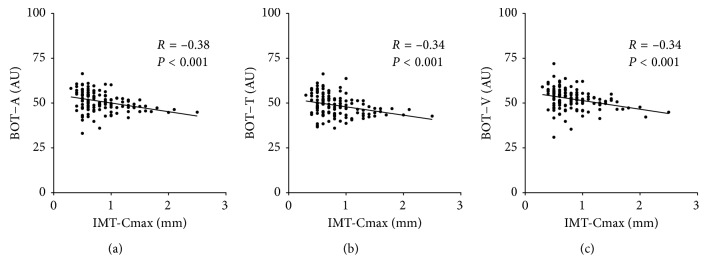
Relationship between IMT-Cmax and BOT. (a) BOT in the overall optic nerve head was negatively correlated with IMT-Cmax (*R*=−0.38, *P* < 0.001). (b) BOT in the optic nerve head tissue area was negatively correlated with IMT-Cmax (*R*=−0.34, *P* < 0.001). (c) BOT in the optic nerve head vascular area was negatively correlated with IMT-Cmax (*R*=−0.34, *P* < 0.001).

MBR-A was negatively correlated with IMT-Cmax (*r* = −0.22, *P*=0.011, Figure 1(a)). MBR-T and MBR-V were also negatively correlated with IMT-Cmax (*r* = −0.20, *P*=0.024, Figure 1(b); *r* = −0.21, *P*=0.016, Figure 1(c)). Finally, BOT-A was negatively correlated with IMT-Cmax (*r* = −0.38, *P* < 0.001, Figure 2(a)). BOT-T and BOT-V were also negatively correlated with IMT-Cmax (*r* = −0.34, *P* < 0.001, Figure 2(b); *r* = −0.34, *P* < 0.001, Figure 2(c)).

Table 2 shows the results of a logistic regression analysis. Age (OR: 0.96; 95% confidence interval (CI): 0.92–0.99; *P*=0.032), diabetes duration (OR: 1.11; 95% CI: 1.03–1.20; *P*=0.006), DBP (OR: 0.93; 95% CI: 0.88–0.99; *P*=0.025), HR (OR: 1.07; 95% CI: 1.02–1.12; *P*=0.004), IMT-Cmax (OR: 8.65; 95% CI: 1.95–38.4; *P*=0.005), and CMT (OR: 1.03; 95% CI: 1.01–1.05; *P*=0.007) were independent factors contributing to the presence of DR, but sex, HbA1c, creatinine, and MBR-A were not (*P*=0.28, 0.74, 0.11, and  0.13, respectively).

**Figure 3:**
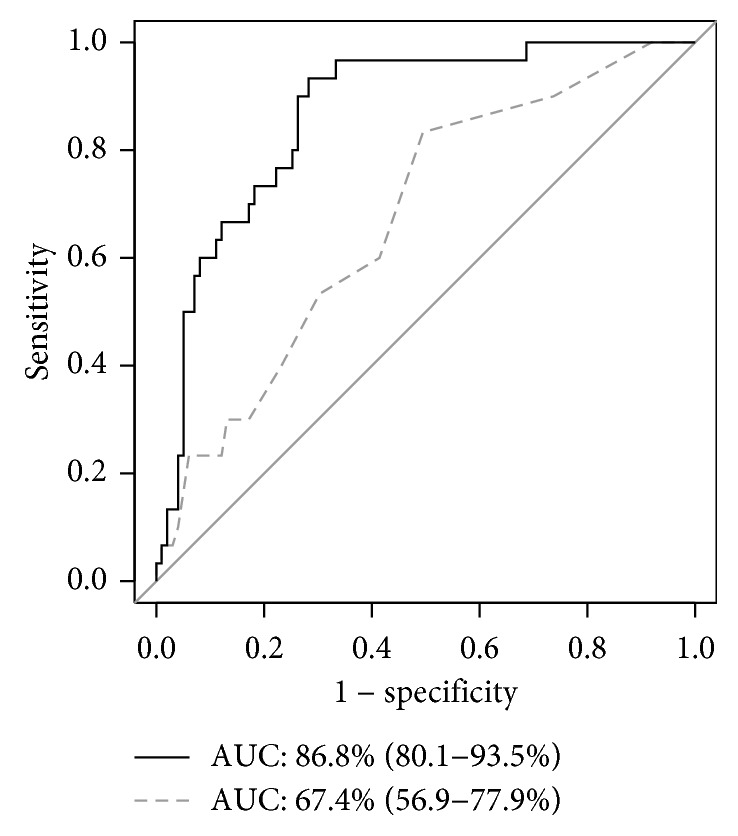
Receiver operating characteristic curves for IMT-Cmax and logistic regression model to predict mild NPDR. The area under the curve (AUC) of IMT-Cmax to predict mild NPDR was 0.67 (95% CI: 0.57–0.78; sensitivity: 83.3%; specificity: 50.5%; shown by the dotted grey line). The AUC for a logistic regression model was 0.87 (95% CI: 0.80–0.94; sensitivity: 93.3%; specificity: 71.7%; shown by the solid black line).

Figure 3 shows the area under the ROC curve (AUC) for IMT-Cmax and for a logistic regression model, representing the power of these factors to predict the presence of mild NPDR. The AUC was 0.67 (95% CI: 0.57–0.78; sensitivity: 83.3%; specificity: 50.5%) for IMT-Cmax and 0.87 (95% CI: 0.80–0.94; sensitivity: 93.3%; specificity: 71.7%) for the logistic regression model.

## 4. Discussion

This study determined the association of ocular and systemic findings, especially IMT-Cmax, in patients with type-2 diabetes and mild NPDR. The results showed that mild NPDR patients had significantly greater diabetes duration, IMT-Cmax, and CMT than NDR patients, while conversely, MBR-A was significantly lower. Furthermore, a logistic regression analysis revealed that IMT-Cmax independently contributed to the presence of mild NPDR. Additionally, Spearman's rank correlation test revealed that IMT-Cmax was negatively correlated to MBR. Thus, increased IMT-Cmax might have a close association with ocular ischemia in type-2 diabetes patients and contributes to the presence of mild NPDR. This finding suggests that IMT-Cmax is a potential early biomarker of mild NPDR.

Generally, duration of diabetes and the level of HbA1c are the most important risk factors for the occurrence and progression of DR [20, 21]. Here, though the NDR and mild NPDR patients had similar levels of HbA1c, the mild NPDR patients had a longer duration of diabetes than the NDR patients. Multiple logistic regression analysis showed that diabetes duration and IMT-Cmax contributed to the presence of mild NPDR, but HbA1c level did not. This finding confirms that diabetes duration is still an important indicator for the occurrence of mild NPDR. It is unclear why HbA1c was not a risk factor in our analysis, but we believe that there are many reasons. In a previous study, we examined an entirely different group of diabetes patients who also showed no significant difference in HbA1c between DR and NDR groups [22]. HbA1c does not reflect blood sugar fluctuations, which cause oxidative stress-derived endothelial damage, and continuous glucose monitoring systems (CGMSs) are therefore important for diabetes control. Furthermore, the mean amplitude of glycemic excursions (MAGE) should also be considered [23]. CGMSs are becoming more common and promise to make it easier for patients to assess and manage their own glycemic variability [24]. It has also been reported that blood sugar fluctuations are reflected more closely by glycated albumin than HbA1c [25, 26]. Thus, parameters other than HbA1c might be the promising markers of mild NPDR.

The current study confirmed that HR was closely associated with the presence of mild NPDR. Several studies have demonstrated that high HR is associated with the risk of DR [27–30]. Generally, high HR reflects the predominance of the sympathetic nervous system, which is associated with the risk of cardiovascular death [31]. Sympathetic nerve activity generally involves the activation of the renin-angiotensin system (RAS). The retina has no functional sympathetic innervation, but the retinal vessels do contain angiotensin receptors. Therefore, microvascular dysfunction due to RAS activation might contribute to the risk of DR in diabetes patients with high HR. Our result showing the association of high HR with mild NPDR is therefore consistent with previous reports on the association between patient background and DR as a microvascular complication [27–30].

Our finding of an association between ultrasound-measured IMT-Cmax and the presence of mild NPDR is particularly interesting; this reinforces several previous reports (Table 3) [11, 32–34]. IMT reflects the thickness of the tunica intima and tunica media, i.e., the innermost artery wall layers, and is clinically used to detect the presence of atherosclerotic disease and to evaluate the progression of atherosclerosis over time [35, 36]. Carotid IMT is associated with type-2 diabetes, and IMT increases in about one-third of diabetes patients with impaired glucose tolerance [37]. Additionally, clinical reports on the relationship between carotid IMT and DR have shown that carotid IMT is higher in patients with PDR than those with NPDR [33]. Carotid plaque levels are also higher in patients with type-2 diabetes and DR than those with NDR [38]. IMT in the current study was an independent factor strongly predicting the presence of mild NPDR, with an odds ratio of about 8. This may be because we used IMT-Cmax, rather than mean IMT. IMT-Cmax has previously reported to be more strongly associated with cardiovascular diseases than mean IMT [39]. Thus, current and past results suggest that increased carotid IMT, which normally reflects thickening of large vessels such as the carotid artery, is also closely associated with the pathogenesis of DR, as well as other diseases associated with alterations in much smaller vessels. Our results reveal that other parameters, including age, duration of diabetes, DBP, heart rate, and CMT, independently contribute to mild NPDR. However, the odds ratio of these parameters was weak (between 0.93 and 1.11), while the adjusted odds ratio of IMT-Cmax was high (more than 8.0). Thus, IMT-Cmax may be the most promising parameter to predict mild NPDR. However, though the discriminative power for mild NPDR of IMT-Cmax by itself was not sufficient (AUC: 0.67), the power of a logistic regression model including all variables was high (AUC: 0.87). We speculate that this may be because DR is a multifactorial disease.

The association between IMT-Cmax and ocular blood flow is another interesting finding of the current study. We found that increased IMT-Cmax reflected reduced MBR in the overall ONH, the tissue area, and the vessel area. This may be due to the accumulation of advanced glycation end products (AGEs), which are known to affect microcirculation in the eye and contribute to DR pathogenesis. Previous research showed that MBR-T had a close association with the level of AGEs in patients with type-2 diabetes and early DR, indicating that the ocular microcirculation could be a source of early biomarkers of DR [40]. Furthermore, in the current study, increased IMT-Cmax reflected reduced BOT in the overall ONH, the tissue area, and the vessel area. BOT represents the full duration at half maximum value of the MBR waveform, and therefore represents the half-duration of a single beat. A high value for BOT shows that the volume of blood flow is high for a long period between beats and that peripheral blood supply is therefore adequate. Changes in BOT in the ONH can reveal early atherosclerotic damage in the ONH [19]. Recently, stiffening of the arteries, represented by the cardio-ankle vascular index, was also reported to be an important contributor to ONH microcirculation [41]. BOT and IMT-Cmax may reflect microvascular and macrovascular atherosclerosis, respectively. The exact reason why BOT did not show a significant difference between the NDR and mild NPDR patients in the current study, even though IMT-Cmax did show a difference, is still unclear. One possible reason is that the choroid, which includes relatively large vessels, is closely involved in DR pathogenesis [42–44]. Taken together with our current finding of parallel associations between macro- and microvascular changes, we believe that macrovascular complications of atherosclerosis occur simultaneously with microvascular ischemia, even during early DR.

Limitations of this study included its use of cross-sectional data, relatively few subjects, and absence of DR-free controls. Nevertheless, we were able to confirm that IMT-Cmax is closely related to LSFG parameters in NDR and mild NPDR, although the underlying mechanisms remain unclear and may be complicated. Indeed, although atherosclerosis likely influences ocular microvascular ischemia, we observed no significant differences in MBR-T, MBR-V, BOT-A, BOT-T, or BOT-V in the NDR and mild NPDR patients. Furthermore, within-day glycemic variability, which we did not evaluate, might also play an important role in the development of DR in type 2 diabetes [45], even though it is minor in type 1 diabetes [46]. Lastly, considering the relatively wide CI for IMT-Cmax, likely due to statistically significant differences in the distribution of the subjects, it is possible that the associations we measured with IMT-Cmax might have been overestimated, leading to an exaggerated OR for IMT-Cmax. Nevertheless, our diabetes subjects were carefully selected and our findings should be reliable. We included only patients with type-2 diabetes in this study, because it is generally considered a different disease than type-1 diabetes, with a distinct pathomechanism. Furthermore, type-2 diabetes has an increasing prevalence worldwide. Finally, we excluded patients who were undergoing hemodialysis, or who had any inflammatory, malignant, or respiratory diseases, giving further strength to our conclusions. Overall, we believe that limiting the focus of this study to type-2 diabetes allowed us to obtain clearer evidence in support of our conclusion.

Thus, the main finding of this study was that mild NPDR patients had significantly higher IMT-Cmax and significantly lower MBR-A than NDR patients. Furthermore, IMT-Cmax was negatively correlated with LSFG ocular blood flow parameters, i.e., MBR-A, MBR-T, MBR-V, BOT-A, BOT-T, and BOT-V. Moreover, among the factors examined in this study, IMT-Cmax was the strongest independent contributor to the presence of mild NPDR. Thus, high IMT-Cmax might be closely related to ocular ischemia in type-2 diabetes and contribute to mild NPDR. IMT-Cmax may therefore be a novel, early source of biomarkers of mild NPDR. Additional investigation should confirm that IMT-Cmax has a causal relationship with diabetic changes in the structure, function, and blood flow of the eye.

## Figures and Tables

**Table 1:** Clinical characteristics of diabetes patients with NDR and mild NPDR.

	NDR	Mild NPDR	*P* value
Number of eyes	99	30	—
Number of patients	99	30	—
Age (years)	54.0 (40.5–64.5)	55.5 (43.0–63.5)	0.676
Sex (M/F)	55/44	20/10	0.384^a^
Duration of DM (years)	6.0 (2.0–11.5)	11.0 (6.3–15.8)	0.004^*∗*^
SBP (mmHg)	128.0 (117.0–140.0)	125.5 (120.0–142.5)	0.670
DBP (mmHg)	79 (72–87)	78 (70–85)	0.370
Heart rate (/min)	72.0 (66.0–79.0)	76.5 (69.5–85.5)	0.670
HbA1c (%)	9.0 (8.0–10.6)	9.6 (8.6–10.4)	0.384
Creatinine (mg/dl)	0.69 (0.55–0.81)	0.71 (0.54–0.84)	0.922
Total cholesterol (mg/dl)	180.0 (153.0–201.5)	178.0 (155.8–209.5)	0.585
IMT-Cmax (mm)	0.6 (0.5–0.9)	0.9 (0.7–1.2)	0.004^*∗*^
VA (logMAR)	−0.079 (−0.18–0)	−0.079 (−0.079–0)	0.183
SE (spherical)	−1.63 (−4.43–−0.25)	−1.81 (−3.19–−0.28)	0.944
IOP (mmHg)	16.0 (14.0–18.0)	15.5 (14.0–18.0)	0.996
LSFG MBR-A (AU)	21.7 (19.0–24.1)	19.1 (16.6–22.7)	0.045^*∗*^
MBR-T (AU)	11.4 (9.9–13.2)	10.7 (9.7–11.4)	0.159
MBR-V (AU)	41.0 (35.4–45.6)	38.6 (34.5–42.0)	0.130
BOT-A	50.6 (47.5–54.6)	51.8 (47.7–54.2)	0.995
BOT-T	48.0 (44.1–52.1)	48.2 (44.7–53.2)	0.653
BOT-V	52.1 (49.1–55.5)	52.3 (49.1–55.3)	0.856
CMT (*µ*m)	236.0 (221.5–250.0)	247.0 (239.3–266.5)	0.003^*∗*^

NDR = no diabetic retinopathy; NPDR = nonproliferative diabetic retinopathy; SBP = systolic blood pressure; DBP = diastolic blood pressure; IMT-Cmax = maximum intima-media thickness in the common carotid artery; VA = visual acuity; SE = spherical equivalent; IOP = intraocular pressure; LSFG = laser speckle flowgraphy; MBR-A = overall mean blur rate; MBR-T = mean blur rate in the tissue area; MBR-V = mean blur rate in the vascular area; BOT-A = overall blowout time; BOT-T = blowout time in the tissue area; BOT-V = blowout time in the vascular area; AU = arbitrary unit; CMT = central macular thickness. Unmarked *P* value: Mann–Whitney U test; ^a^chi-square test. Continuous variables: median (interquartile range). ^*∗*^*P* < 0.05.

**Table 2:** Multiple logistic regression analysis of factors independently contributing to mild NPDR.

Variables		Adjusted OR (95% CI)	*P* value
Dependent	Independent		
Presence of mild NPDR	Age (years)	0.96 (0.92–0.99)	0.032
	Sex (M/F)	1.99 (0.58–6.82)	0.276
	HbA1c (%)	1.05 (0.80–1.38)	0.740
	Duration of DM (year)	1.11 (1.03–1.20)	0.006
	DBP (mmHg)	0.93 (0.88–0.99)	0.025
	Heart rate (/min)	1.07 (1.02–1.12)	0.004
	IMT-Cmax (mm)	8.65 (1.95–38.4)	0.005
	CMT (*µ*m)	1.03 (1.01–1.05)	0.007
	Creatinine (mg/dl)	0.12 (0.01–1.64)	0.110
	MBR-A (AU)	0.92 (0.83–1.02)	0.134

Nagelkerke's *R*^2^ = 0.40. OR = odds ratio; NPDR = nonproliferative diabetic retinopathy; DM = diabetic mellitus; DBP = diastolic blood pressure; IMT-Cmax = maximum intima-media thickness of common carotid artery; CMT = central macular thickness; MBR-A = mean blur rate in the overall optic nerve head.

**Table 3:** Comparison of previous reports.

Studies	Subjects	Number of eyes	95% CI^*∗*^	Major findings of IMT
Momeni et al. [11]	DR vs. NDR	154	DR: 0.95 (0.91–0.99) NDR: 0.73 (0.71–0.75)	IMT-Cmax was significantly higher in DR than in NDR

Kocaoglu et al. [32]	DR vs. NDR vs. control	85	DR: 0.9 (0.84–0.96) NDR: 0.8 (0.74–0.86) Control: 0.7 (0.65–0.75)	IMT was significantly higher in DR than in NDR, but showed no significant difference between NDR and controls

Saif et al. [33]	PDR vs. NPDR	140	PDR: 1.094 (1.062–1.126) NPDR: 0.842 (0.810–0.874)	Mean IMT was significantly higher in PDR than in NPDR

Rema et al. [34]	DR vs. NDR	590	DR: 0.93 (0.86–1.00) NDR: 0.85 (0.83–0.87)	Mean IMT was significantly higher in DR than in NDR

Current study	NDR vs. Mild NPDR	129	Mild NPDR: 1.00 (0.83–1.17) NDR: 0.77 (0.70–0.84)	IMT-Cmax was significantly higher in mild NPDR than in NDR

DR = diabetic retinopathy; NPDR = nonproliferative diabetic retinopathy; CI = confidence interval; IMT = intima-media thickness. ^*∗*^95% CI of each previous report was calculated from mean, standard deviation, and sample size shown in the paper.

## Data Availability

The data used to support the findings of this study cannot be made freely available. Requests for access to these data should be made to Dr. Ichinohasama (email: ichinohasama-kohei@oph.med.tohoku.ac.jp).

## References

[1] Ruta L. M., Magliano D. J., LeMesurier R., Taylor H. R., Zimmet P. Z., Shaw J. E. (2013). Prevalence of diabetic retinopathy in Type 2 diabetes in developing and developed countries. *Diabetic Medicine*.

[2] Whiting D. R., Guariguata L., Weil C., Shaw J. (2011). IDF diabetes atlas: global estimates of the prevalence of diabetes for 2011 and 2030. *Diabetes Research and Clinical Practice*.

[3] Shimura M., Nakazawa T., Yasuda K. (2008). Comparative therapy evaluation of intravitreal bevacizumab and triamcinolone acetonide on persistent diffuse diabetic macular edema. *American Journal of Ophthalmology*.

[4] Schoenberger S. D., Miller D. M., Riemann C. D. (2011). Outcomes of 25-gauge pars plana vitrectomy in the surgical management of proliferative diabetic retinopathy. *Ophthalmic Surgery, Lasers, and Imaging*.

[5] Virgili G., Parravano M., Menchini F., Evans J. R. (2014). Anti-vascular endothelial growth factor for diabetic macular oedema. *Cochrane Database of Systematic Reviews*.

[6] Kwon J. W., Jee D., La T. Y. (2017). Neovascular glaucoma after vitrectomy in patients with proliferative diabetic retinopathy. *Medicine*.

[7] Safi H., Safi S., Hafezi-Moghadam A., Ahmadieh H. (2018). Early detection of diabetic retinopathy. *Survey of Ophthalmology*.

[8] Ito A., Kunikata H., Yasuda M. (2018). The relationship between peripheral nerve conduction velocity and ophthalmological findings in type 2 diabetes patients with early diabetic retinopathy. *Journal of Ophthalmology*.

[9] Ando T., Okada S., Niijima Y. (2010). Impaired glucose tolerance, but not impaired fasting glucose, is a risk factor for early-stage atherosclerosis. *Diabetic Medicine*.

[10] Gong W., Lu B., Yang Z. (2009). Early-stage atherosclerosis in newly diagnosed, untreated type 2 diabetes mellitus and impaired glucose tolerance. *Diabetes & Metabolism*.

[11] Momeni A., Dyani M. A., Ebrahimi E., Sedehi M., Naderi A. (2015). Association of retinopathy and intima media thickness of common carotid artery in type 2 diabetic patients. *Journal of Research in Medical Sciences*.

[12] Rina M., Shiba T., Takahashi M., Hori Y., Maeno T. (2015). Pulse waveform analysis of optic nerve head circulation for predicting carotid atherosclerotic changes. *Graefe’s Archive for Clinical and Experimental Ophthalmology*.

[13] Early Treatment Diabetic Retinopathy Study Research Group (1991). Grading diabetic retinopathy from stereoscopic color fundus photographs--an extension of the modified airlie house classification. ETDRS report number 10. *Ophthalmology*.

[14] Early Treatment Diabetic Retinopathy Study Research Group (1991). Early Treatment Diabetic Retinopathy Study design and baseline patient characteristics. ETDRS report number 7. *Ophthalmology*.

[15] Ogawa Y., Uchigata Y., Iwamoto Y. (2009). Progression factors of carotid intima-media thickness and plaque in patients with long-term, early-onset type 1 diabetes mellitus in Japan: simultaneous comparison with diabetic retinopathy. *Journal of Atherosclerosis and Thrombosis*.

[16] Isono H., Kishi S., Kimura Y., Hagiwara N., Konishi N., Fujii H. (2003). Observation of choroidal circulation using index of erythrocytic velocity. *Archives of Ophthalmology*.

[17] Tamaki Y., Araie M., Kawamoto E., Eguchi S., Fujii H. (1995). Non-contact, two-dimensional measurement of tissue circulation in choroid and optic nerve head using laser speckle phenomenon. *Experimental Eye Research*.

[18] Shiba T., Takahashi M., Hori Y., Maeno T., Shirai K. (2012). Optic nerve head circulation determined by pulse wave analysis is significantly correlated with cardio ankle vascular index, left ventricular diastolic function, and age. *Journal of Atherosclerosis and Thrombosis*.

[19] Shiba T., Takahashi M., Hori Y., Maeno T. (2012). Pulse-wave analysis of optic nerve head circulation is significantly correlated with brachial-ankle pulse-wave velocity, carotid intima-media thickness, and age. *Graefe’s Archive for Clinical and Experimental Ophthalmology*.

[20] Klein R., Klein B. E., Moss S. E., Davis M. D., DeMets D. L. (1984). The Wisconsin epidemiologic study of diabetic retinopathy. *Archives of Ophthalmology*.

[21] Klein R., Klein B. E., Moss S. E., Davis M. D., DeMets D. L. (1984). The Wisconsin epidemiologic study of diabetic retinopathy. *Archives of Ophthalmology*.

[22] Yasuda M., Shimura M., Kunikata H. (2014). Relationship of skin autofluorescence to severity of retinopathy in type 2 diabetes. *Current Eye Research*.

[23] Service F. J., Molnar G. D., Rosevear J. W., Ackerman E., Gatewood L. C., Taylor W. F. (1970). Mean amplitude of glycemic excursions, a measure of diabetic instability. *Diabetes*.

[24] Monnier L., Colette C., Owens D. R. (2008). Glycemic variability: the third component of the dysglycemia in diabetes. Is it important? How to measure it?. *Journal of Diabetes Science and Technology*.

[25] Yoshiuchi K., Matsuhisa M., Katakami N. (2008). Glycated albumin is a better indicator for glucose excursion than glycated hemoglobin in type 1 and type 2 diabetes. *Endocrine Journal*.

[26] Takahashi S., Uchino H., Shimizu T. (2007). Comparison of glycated albumin (GA) and glycated hemoglobin (HbA1c) in type 2 diabetic patients: usefulness of GA for evaluation of short-term changes in glycemic control. *Endocrine Journal*.

[27] Zhang G., Chen H., Chen W., Zhang M. (2017). Prevalence and risk factors for diabetic retinopathy in China: a multi-hospital-based cross-sectional study. *British Journal of Ophthalmology*.

[28] Bulum T., Blaslov K., Duvnjak L. (2013). Resting heart rate is associated with nonproliferative retinopathy in normoalbuminuric type 1 diabetic patients. *Journal of Clinical Hypertension*.

[29] Hillis G. S., Hata J., Woodward M. (2012). Resting heart rate and the risk of microvascular complications in patients with type 2 diabetes mellitus. *Journal of the American Heart Association*.

[30] Imano E., Miyatsuka T., Motomura M. (2001). Heart rate elevation and diabetic retinopathy in patients with type 2 diabetes mellitus and normoalbuminuria. *Diabetes Research and Clinical Practice*.

[31] Anan F., Takayuki M., Takahashi N. (2009). Diabetic retinopathy is associated with insulin resistance and cardiovascular autonomic dysfunction in type 2 diabetic patients. *Hypertension Research*.

[32] Kocaoglu I., Kocaoglu E., Arslan U. (2016). Relationship between retinopathy and asymptomatic atherosclerosis determined by measurement of carotid intima-media thickness in patients with type 2 diabetes mellitus. *Archives of the Turkish Society of Cardiology*.

[33] Saif A., Karawya S., Abdelhamid A. (2015). Retinopathy is a strong determinant of atherosclerosis in type 2 diabetes: correlation with carotid intima media thickness. *Endocrine Practice*.

[34] Rema M., Mohan V., Deepa R., Ravikumar R. (2004). Association of carotid intima-media thickness and arterial stiffness with diabetic retinopathy: the Chennai Urban Rural Epidemiology Study (CURES-2). *Diabetes Care*.

[35] de Groot E., van Leuven S. I., Duivenvoorden R. (2008). Measurement of carotid intima-media thickness to assess progression and regression of atherosclerosis. *Nature Clinical Practice Cardiovascular Medicine*.

[36] O’Leary D. H., Bots M. L. (2010). Imaging of atherosclerosis: carotid intima-media thickness. *European Heart Journal*.

[37] Brohall G., Oden A., Fagerberg B. (2006). Carotid artery intima-media thickness in patients with type 2 diabetes mellitus and impaired glucose tolerance: a systematic review. *Diabetic Medicine*.

[38] Alonso N., Traveset A., Rubinat E. (2015). Type 2 diabetes-associated carotid plaque burden is increased in patients with retinopathy compared to those without retinopathy. *Cardiovascular Diabetology*.

[39] Bots M. L., Evans G. W., Riley W. A., Grobbee D. E. (2003). Carotid intima-media thickness measurements in intervention studies. *Stroke*.

[40] Hashimoto K., Kunikata H., Yasuda M. (2016). The relationship between advanced glycation end products and ocular circulation in type 2 diabetes. *Journal of Diabetes and its Complications*.

[41] Shiba T., Takahashi M., Matsumoto T., Shirai K., Hori Y. (2016). Arterial stiffness shown by the cardio-ankle vascular index is an important contributor to optic nerve head microcirculation. *Graefe’s Archive for Clinical and Experimental Ophthalmology*.

[42] Wang J. C., Laíns I., Providência J. (2017). Diabetic choroidopathy: choroidal vascular density and volume in diabetic retinopathy with swept-source optical coherence tomography. *American Journal of Ophthalmology*.

[43] Lutty G. A. (2017). Diabetic choroidopathy. *Vision Research*.

[44] Shen Z. J., Yang X. F., Xu J. (2017). Association of choroidal thickness with early stages of diabetic retinopathy in type 2 diabetes. *International Journal of Ophthalmology*.

[45] Hsu C.-R., Chen Y.-T., Sheu W. H.-H. (2015). Glycemic variability and diabetes retinopathy: a missing link. *Journal of Diabetes and its Complications*.

[46] Lachin J. M., Bebu I., Bergenstal R. M. (2017). Association of glycemic variability in type 1 diabetes with progression of microvascular outcomes in the diabetes control and complications trial. *Diabetes Care*.

